# Physical Pretreatments of Cassava Chips Influenced Chemical Composition, Physicochemical Properties, and In Vitro Digestibility in Animal Models

**DOI:** 10.3390/ani14060908

**Published:** 2024-03-15

**Authors:** Suriyanee Takaeh, Sukanya Poolthajit, Waraporn Hahor, Nutt Nuntapong, Wanwisa Ngampongsai, Karun Thongprajukaew

**Affiliations:** 1 Division of Health and Applied Sciences, Faculty of Science, Prince of Songkla University, Songkhla 90110, Thailand; suriyanee.ta@gmail.com (S.T.); sukanyapoolthajit@rmutl.ac.th (S.P.); waraporn.hahor@gmail.com (W.H.); 2 Kidchakan Supamattaya Aquatic Animal Health Research Center, Aquatic Science and Innovative Management Division, Faculty of Natural Resources, Prince of Songkla University, Songkhla 90110, Thailand; nutt.n@psu.ac.th; 3 Animal Production Innovation and Management Division, Faculty of Natural Resources, Prince of Songkla University, Songkhla 90110, Thailand; wanwisa.n@psu.ac.th

**Keywords:** aquatic animal, feed ingredient, nutritive value, poultry, pretreatment, proximate composition, ruminant

## Abstract

**Simple Summary:**

To improve the quality of feedstuff for use as animal feed, four alternative pretreatment methods (extrusion, microwave irradiation, gamma irradiation, or NaOH hydrolysis) were applied for cassava chips. The proximate chemical compositions and nutritive profiles were significantly altered due to the pretreatment methods (*p* < 0.05), as well as the physicochemical properties in aspects to enhance enzymatic hydrolysis. Based on in vitro carbohydrate digestibility, microwave irradiation was suitable for improving nutrient bioavailability in the aquatic animal model, Nile tilapia, while microwaving, followed by extrusion or gamma irradiation, was suitable for the poultry model, broiler chicken. However, no differences were observed for the ruminant model. Our in vitro screening investigations support the use of pretreated cassava chips for animal feeding, especially for fish and broiler.

**Abstract:**

Physical pretreatment procedures can significantly influence the quality of food and feed raw materials. To increase the ability to be digested in animals, cassava chips were pretreated by four alternative methods (extrusion, microwave irradiation, gamma irradiation, or NaOH hydrolysis), and then the chemical composition, physicochemical properties, and in vitro digestibility of the pretreated samples were assessed and compared with unprocessed cassava chips (control). The chemical compositions (crude protein, ether extract, neutral detergent fiber, acid detergent fiber, ash, non-fiber carbohydrate, and gross energy) were significantly altered due to the pretreatment methods (*p* < 0.05). The nutritive profile was qualitatively changed when assessed through Fourier-transform infrared spectroscopy. Some physicochemical properties in association with enzymatic hydrolysis, which include pH, water solubility, water absorption capacity, thermal properties (differential scanning calorimetry), diffraction pattern (X-ray diffractometry), and microstructure (scanning electron microscopy), were significantly changed. In vitro carbohydrate digestibility based on digestive enzyme extracts from Nile tilapia (*Oreochromis niloticus*) suggests the microwaving method for cassava chips preparation, while microwaving, followed by extrusion or gamma irradiation, was suggested for broiler (*Gallus gallus domesticus*). There were no differences in the pepsin-cellulase digestibility values tested for the ruminant model. These findings suggest the use of pretreated cassava chips in animal feeding.

## 1. Introduction

For its drought tolerance, cassava is cultivated in Africa as food, while, in Asia, its products are mainly used as animal feed [1]. In general, cassava contains >90% carbohydrates and ~3% protein in its dry matter, making it a high-energy source of feedstuff [2]. They provide a desirable in vitro ruminal fermentation performance and organic matter degradability [3]. Additionally, cassava could be incorporated into the feed of monogastric animals, such as poultry and fish [4,5]. However, cassava has inherent differences in terms of solubility, fermentability, and digestibility, as compared to other carbohydrate-rich feedstuffs. Because the crystalline structure and intact granules are difficult to hydrolyze [6], a process that reduces the crystalline structure to increase efficiency and the ability to be digested in animals should be of interest.

Many previous studies have attempted to improve the quality of raw materials in livestock feed. For cassava chips, sun-drying of around 3 to 6 days, followed by a few days’ storage, is a practical protocol before shipment to the feed manufacturer [7]. For other raw materials containing a large portion of carbohydrates, the extrusion process creates frictional heating under high pressure which stimulates the gelatinization of starch [8]. Moreover, in the case of cassava, this process also eliminates the risk of hydrocyanic acid (HCN) toxicity to the animals [7]. Microwaving and gamma irradiation can also improve food and feed ingredient qualities by altering some physicochemical properties to enhance enzymatic hydrolysis [9,10,11,12]. In addition, alkali treatment can break down strong structures and cuticle protein, resulting in increased solubility and degradation [13]. These four pretreatment methods are practical for feed preparation, have a less-time operation, and are acceptable in the feed industry. Moreover, physical pretreatments probably reduce contamination with unfavorable micro-organisms as compared to biological pretreatments. Not only does the chemical composition change, but these pretreatments also improve the molecular composition and increase the enzyme space in animal digestibility [14,15]. However, a comparative investigation of these physical methods in improving the quality of cassava chips is still lacking.

Pretreatment procedures can significantly influence the physicochemical properties of starch, thereby impacting its quality [16]. The desirable effects from material pretreatments can be traced by properties in aspects to enhance enzymatic hydrolysis in vitro, such as pH, water solubility, water absorption capacity (WAC), thermal properties, diffraction pattern and relative crystallinity, and microstructure [17,18,19]. In vitro digestibility can be applied to assess bioavailability in animals. This practical, inexpensive technique is comparatively rapid and can perform multiple reactions simultaneously, effectively predicting in vivo growth trials [17,20]. These observed parameters might suggest a suitable protocol for the pretreatment of cassava chips prior to use in animal feeds.

Therefore, the objective of this study was to investigate the effects of pretreatment methods on the proximate chemical composition, physicochemical properties, and in vitro digestibility of cassava chips. The in vitro digestibility based on digestive enzymes from Nile tilapia (*Oreochromis niloticus*) and broiler (*Gallus gallus domesticus*) was treated as representative for the respective groups of aquatic animals and poultry. Concurrently, the pepsin-cellulase technique, mimicking the reactions that occur in the digestive tract, was used for the ruminant group. The findings from the current study can be applied to improve the quality of cassava chips for a wide range of animal groups.

## 2. Materials and Methods

### 2.1. Cassava Chip Pretreatments

The cassava chips were obtained from an industrial factory in Songkhla province, Thailand. The unprocessed cassava chips were used as a control where pretreatment methods were varied (extrusion, microwave irradiation, gamma irradiation, and NaOH hydrolysis). The samples of powdered cassava chips were steamed in conditions of 82 °C for 45 min, and then pelleted through a ring die of 4.0 mm pellet mill (Model 1112-4; California Pellet Mill Co., Crawfordsville, IN, USA). The extrudates were broken into pieces and then dried at 50 °C for 2 h. To obtain a microwave-irradiated cassava chip, 300 g of dried samples were placed in a closed spherical plastic box (23 cm diameter × 10.5 cm height), mixed with distilled water (2:1 *w*/*v*), and then irradiated in a microwave oven (MW71B; Samsung, Kuala Lumpur, Malaysia) at 700 W for 11 min [11]. The gamma irradiation pretreatment was conducted at the Institute of Nuclear Technology, Thailand. A Co from carrier-type gamma irradiation (JS 8900 IR-155, MDS Nordion, Ottawa, ON, Canada) provided a dose of 30 kGy for the samples [21]. The NaOH alkaline treatment was conducted as described by De Campeneere et al. [22] with slight modifications. The raw cassava chips were treated with 3.5% of 35% (*w*/*v*) of NaOH for 15 min, and then treated samples and residual NaOH were loosely packed into one jar per treatment. After a reopened time, the mixed samples were spread on a carpet for 48 h in room condition before drying in the oven at 60 °C for 48 h. All the samples were prepared in triplicate.

### 2.2. Preparation of Pretreated Samples

A freeze dryer (Delta 2–24 LSC, Martin Christ Gefriertrocknungsanlagen GmbH, Osterode am Harz, Germany) was used to eliminate moisture for 24 h. The freeze-dried samples were ground, sieved, packed in polyethylene bags, and then stored at 4 °C until analysis of chemical composition, physicochemical properties, and in vitro digestibility.

### 2.3. Proximate Chemical Compositions and Nutritive Profiles

Dry matter (DM), crude protein (CP), ether extract (EE), and ash contents were analyzed according to standard methods of the AOAC [23]. Neutral detergent fiber (NDF) and acid detergent fiber (ADF) were analyzed according to the method of Van Soest et al. [24]. Non-fiber carbohydrate (NFC, %) was calculated from 100 − (% CP + % EE + % NDF + % ash). Gross energy (GE) was quantified using an automatic adiabatic bomb calorimeter (AC500; LecO Corp., St. Joseph, MI, USA).

Fourier-transform infrared spectrometer (FTIR8400s; Shimadzu, Kyoto, Japan) was used to qualitatively describe the change in nutritive values due to pretreatment methods. The technique and program were run as described by Hahor et al. [17] and Thongprajukaew et al. [18] with some modifications. The 2 mg of powdered samples were mixed with 200 mg of KBr in a vacuum dryer for 24 h, and then the mixture was uniformly ground and pressed into a tablet. Spectra from FTIR were acquired in the mid-IR range (4000 to 400 cm^−1^) at a resolution of 4 cm^−1^. The IR spectra were processed using OMNIC 8.0 Software (Nicolet Analytical Instruments, Madison, WI, USA), and, after baseline correction of the original map, the sample and control spectra were compared.

### 2.4. Physicochemical Properties

#### 2.4.1. pH

Alteration of pH was assessed according to the method as described by Sokhey and Chinnaswamy [25]. Freeze-dried samples of 0.25 g were mixed with 6.25 mL of distilled water, agitated for 10 min, and then monitored by using a pH meter.

#### 2.4.2. Water Solubility

The solubility of unprocessed and pretreated samples was determined according to the method of Chung et al. [26]. Briefly, one gram of powdered cassava chips was mixed with 10 mL of water, gently stirred for 1 h, and centrifuged at 1500× *g* for 10 min. The obtained supernatant was oven-dried at 60 °C for 48 h and weighed. The solubility of the sample was calculated from the ratio between weight of dissolved solids in the supernatant and weight of dry solids in the original sample.

#### 2.4.3. Water Absorption Capacity

Water absorption capacity (WAC) was determined as described by Jitngarmkusol et al. [27]. Two grams of cassava chip samples were mixed with 5 mL of distilled water, allowed to situate at 25 °C for 30 min, and centrifuged at 2000× *g* for 10 min. The supernatant was collected and weighed. All values are expressed as g water g sample^−1^.

#### 2.4.4. Differential Scanning Calorimeter

The onset (T_o_), peak (T_p_) and conclusion (T_c_) temperatures, melting temperature range (T_c_ − T_o_), and transition enthalpy (∆H) were determined with a differential scanning calorimeter (DSC7; Perkin Elmer, Waltham, MA, USA). The technique and program were run as described by Hahor et al. [17] and Thongprajukaew et al. [18] with some modifications. Approximately 3 mg of a sample were placed in an aluminum pan, sealed, allowed to equilibrate at room temperature for 1 h, and then heated. The temperature program was run from 40 to 400 °C at a rate of 5 °C min^−1^.

#### 2.4.5. Diffraction Pattern and Relative Crystallinity

X-ray diffractometer (X′ Pert MPD, Philips, The Netherlands) was used to characterize the diffraction patterns and relative crystallinity of unprocessed and pretreated samples of cassava chips. The technique and program were run as described by Hahor et al. [17] and Thongprajukaew et al. [18] with some modifications. The diffraction angles were run from 4 to 40° (2θ), 40 kV, and 30 mA. The measurements of the total area under the curve and the area under each prominent peak were used for estimating the relative crystallinity as follows: Crystallinity (%) = (Area under peaks/total area) × 100.

#### 2.4.6. Microstructure

The microstructure of samples was photographed using a scanning electron microscope (Quanta 400; FEI, Brno, Czech Republic). The technique and program were run as described by Hahor et al. [17] and Thongprajukaew et al. [18] with some modifications. The dried samples were placed on aluminum stubs using double-sided adhesive tape and coated with gold. The accelerating voltage was set at 20 kV. The surface microstructure was recorded and imaged at the magnifications 250× and 1500×.

### 2.5. In Vitro Digestibility Screening

#### 2.5.1. Aquatic Animal and Poultry Models

Three specimens of four-month-old Nile tilapia (*O. niloticus*) and 42-day-old broiler (*G. gallus domesticus*) were obtained from a private farm within the Faculty of Natural Resources, Prince of Songkla University. The animal protocols were approved by the Institutional Animal Care and Use Committees (Code 2022-Sci11-018, approval date 1 June 2022). The fish were fasted for 24 h, and then were sacrificed by chilling in ice. The specimens from broiler were collected at a commercial slaughterhouse during the evisceration process. The intestinal samples were removed on ice, partially dissected, and mixed with 0.2 M phosphate buffer (pH 8 and pH 7, respectively) at a proportion of 1:3 *w*/*v*. The tissues were homogenized using a micro-homogenizer (THP-220; Omni International, Kennesaw, GA, USA) for 20 s, and then centrifuged at 15,000× *g* for 30 min at 4 °C. The supernatant was collected and dialyzed overnight against extraction buffer.

The in vitro digestibility reaction was performed as described by Thongprajukaew et al. [28]. Five milligrams of each dried cassava chip were mixed with 10 mL of 50 mM Na_2_HPO_4_-NaH_2_PO_4_ buffer (pH 8), 50 µL of 0.5% chloramphenicol, and 125 µL of dialyzed crude enzyme extract. The mixtures were incubated at 25 °C for 24 h under 200 rpm agitation. The in vitro protein digestibility was determined by measuring the increase of liberated reactive amino groups of cleaved peptides at 420 nm. The in vitro carbohydrate digestibility was determined by measuring the increase of reducing sugar at 540 nm. The linear ranges of *DL*-alanine and maltose, respectively, were used for comparison. The digestibility values were calculated and are expressed as mmol *DL*-alanine equivalent g sample^−1^ and µmol maltose g sample^−1^, respectively.

#### 2.5.2. Ruminant Model

The pepsin-cellulase digestibility technique was performed according to De Boever et al. [29], and triplicate samples were obtained from different incubation runs. Three hundred milligrams of cassava chip samples were weighed, and placed in a glass filter-crucible, followed by adding 30 mL of pepsin-hydrochloric acid solution at 40 °C. The crucible was incubated at 40 °C for 24 h and shaken every 5 h. After that, they were placed in a water bath at 80 °C for 45 min, after which the solution was aspirated and the residue was rinsed with distilled water. At 40 °C, cellulase-buffer was added into 30 mL of solution and incubated for 24 h and occasionally shaken every 5 h. The solution was aspirated and the residue was washed with distilled water at 40 °C. The digested portion was dried at 103 °C to determine the DM value, while the residue was burned at 550 °C to determine cellulase organic matter solubility (COMS). The calculation is as follows: COMS (%) = (W_o_ − W_t_) × 100, where W_o_ and W_t_ are organic matter (OM) incubated and OM after incubation stages, respectively. Digestible organic matter (DOM) and metabolizable energy (ME) of cassava chips was calculated as follows: DOM (%) = 0.973 × COMS − 2.49, and ME (mJ kg DM^−1^) = 0.150 × COMS − 0.214 × EE − 0.99.

### 2.6. Statistical Analysis

A completely randomized design (CRD) was formulated, comprising five alternative treatments and triplicate observation. All received data are reported as mean ± SEM. Normality and homoscedasticity of data were checked prior to statistical analysis. Differences among means between treatment groups were subjected to one-way analysis of variance using Tukey’s range test as a post hoc test. The hypothesized condition was rejected if the *p*-value was 0.05 or below.

## 3. Results

### 3.1. Chemical Compositions of Unprocessed and Pretreated Cassava Chips

The effects of pretreatments on the proximate chemical composition of cassava chips are illustrated in Table 1. Based on the DM basis, the composition of OM, CP, EE, NDF, ADF, ash, and NFC were significantly different (*p* < 0.05). Improved CP was observed in extruded cassava chips, and vice versa for microwave-irradiated and NaOH-treated samples (*p* < 0.05). All pretreatment methods, except for gamma irradiation, decreased EE significantly (*p* < 0.05). Approximately doubled values of NDF was observed in extruded samples, while significantly decreased values were found in gamma-irradiated and NaOH-treated groups (*p* < 0.05). All pretreatments showed significantly reduced ADF (*p* < 0.05). On the other hand, these methods increased the amounts of ash significantly (*p* < 0.05), except for cassava chips exposed to gamma irradiation. Alteration in these constituents caused significantly increased (decreased) NFC in gamma-irradiated and NaOH-treated groups (extruded and microwave-irradiated groups), and increased (decreased) GE in extruded (NaOH-treated) cassava chips (*p* < 0.05).

### 3.2. Nutritive Profiles of Unprocessed and Pretreated Cassava Chips

**Figure 1:**
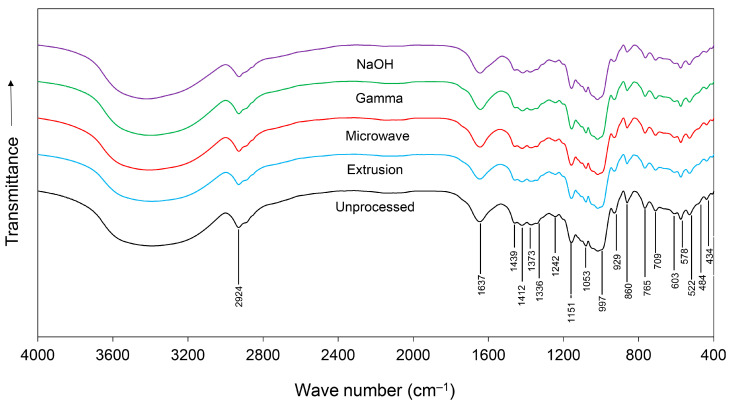
FTIR spectra of unprocessed, extruded, microwave-irradiated, gamma-irradiated, and NaOH-treated cassava chips.

The FTIR spectra of unprocessed and pretreated cassava chips are shown in Figure 1. Overall, the spectra of samples had very similar shapes and peak positions but differed in intensity across the spectral region, which indicated that the samples had the same composition but varied in amounts. Within a range from 4000 to 400 cm^−1^, nineteen bands (2924, 1637, 1439, 1412, 1373, 1336, 1242, 1151, 1053, 997, 929, 860, 765, 709, 603, 578, 522, 484, and 434 cm^−1^) were observed indicating changes in nutritive profiles, specifically proteins, lipids, and carbohydrates (Table 2).

Changes in protein characteristics were indicated by *v*_s_ (C=O) stretching of amide I at 1637 cm^−1^ [31], δ_as_ (CH_3_) bending of methyl at 1439 and 1412 cm^−1^ [32], *v*_s_ (COO-) stretching of amino acid salt at 1373 cm^−1^ [34], and N(C=O) stretching and (C-OH) bending of deprotonated amino acid at 1242 cm^−1^ [35]. Modifications of lipid profiles were indicated by *v*_as_ (CH_2_) stretching of methylene at 2924 cm^−1^ [29,30], δ_as_ (CH_2_) bending of methyl at 1439 and 1412 cm^−1^ [29,30], *v*_as_ (PO_4_^3−^) P-O asymmetric stretching of lipids at 860 cm^−1^ [37], and (CH^2-^), C-H rocking of lipids at 709 cm^−1^ [30].

Modifications of carbohydrates were indicated by *v*_s_ (C=O) stretching vibrations of carboxylate at 1412, 1373, and 1336 cm^−1^ [33] and *v* (C-O-C) stretching of polysaccharide at 1151, 1053, and 997 cm^−1^ [36]. Changes to alkene at 929 cm^−1^ [34], amide at 522 cm^−1^ [39], and inorganic compounds at 765, 603, 578, 484, and 434 cm^−1^ [32,38,40] were also observed.

### 3.3. Physicochemical Properties of Unprocessed and Pretreated Cassava Chips

#### 3.3.1. pH

The pretreatment methods had a significant effect on the pH of cassava chips (*p* < 0.05, Table 3). The highest values were observed in NaOH-treated cassava chips, followed by microwave irradiation (*p* < 0.05). Treatment with gamma irradiation led to a significantly reduced pH value relative to unprocessed cassava chips (*p* < 0.05).

#### 3.3.2. Water Solubility and WAC

Water solubility was significantly higher for the extrusion, followed by NaOH pretreatment (*p* < 0.05, Table 3). Unchanged solubility was observed in the microwave-irradiated and gamma-irradiated groups (*p* > 0.05). Decreased WAC was only observed in the extruded group (*p* < 0.05, Table 3).

#### 3.3.3. Thermal Transition Properties

The DSC thermograms and corresponding thermal transition temperatures (T_o_, T_p_, and T_c_) and enthalpy change (∆H) of the samples are shown in Table 4. Two peaks of thermal response were observed, except for in cassava chips pretreated by NaOH. In general, peak I indicated nutrients absorbing heat energy within a temperature range of 46–157 °C, while peak II was observed within a range of 259–281 °C. At lower temperatures, pretreatment methods did not affect all transition temperatures (*p* > 0.05) but significantly decreased the ∆H observed in pretreatment groups (*p* < 0.05), except for microwave irradiation. At higher temperatures, microwave-irradiated samples had unchanged transition temperatures (*p* > 0.05), but ∆H decreased significantly (*p* < 0.05). In contrast, altered transition temperatures (*p* < 0.05) but maintained ∆H were found in the extruded and gamma-irradiated samples (*p* > 0.05).

#### 3.3.4. Diffraction Pattern

**Figure 2:**
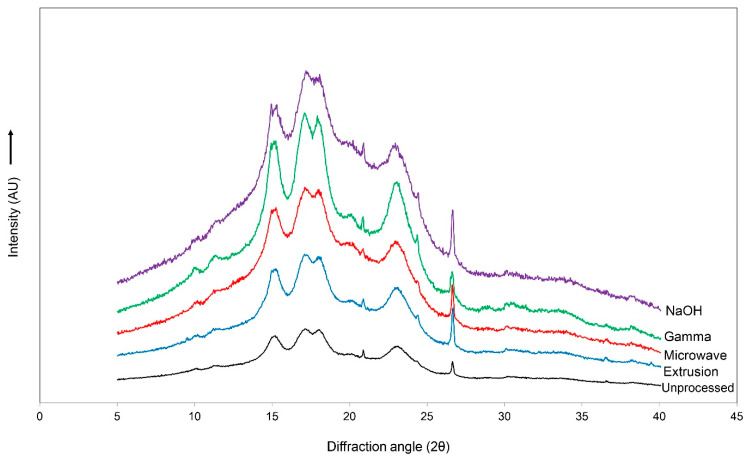
Diffraction patterns of unprocessed, extruded, microwave-irradiated, gamma-irradiated, and NaOH-treated cassava chips.

Similar diffraction patterns of the main peaks were observed at 15.1, 17.1, 18.0, 20.8, 22.9, 24.4, and 26.6° (2θ) (Figure 2). The relative crystallinity was not caused by the pretreatment methods (*p* > 0.05); the values are within the range of 24.9–26.9%.

#### 3.3.5. Microstructure

**Figure 3:**
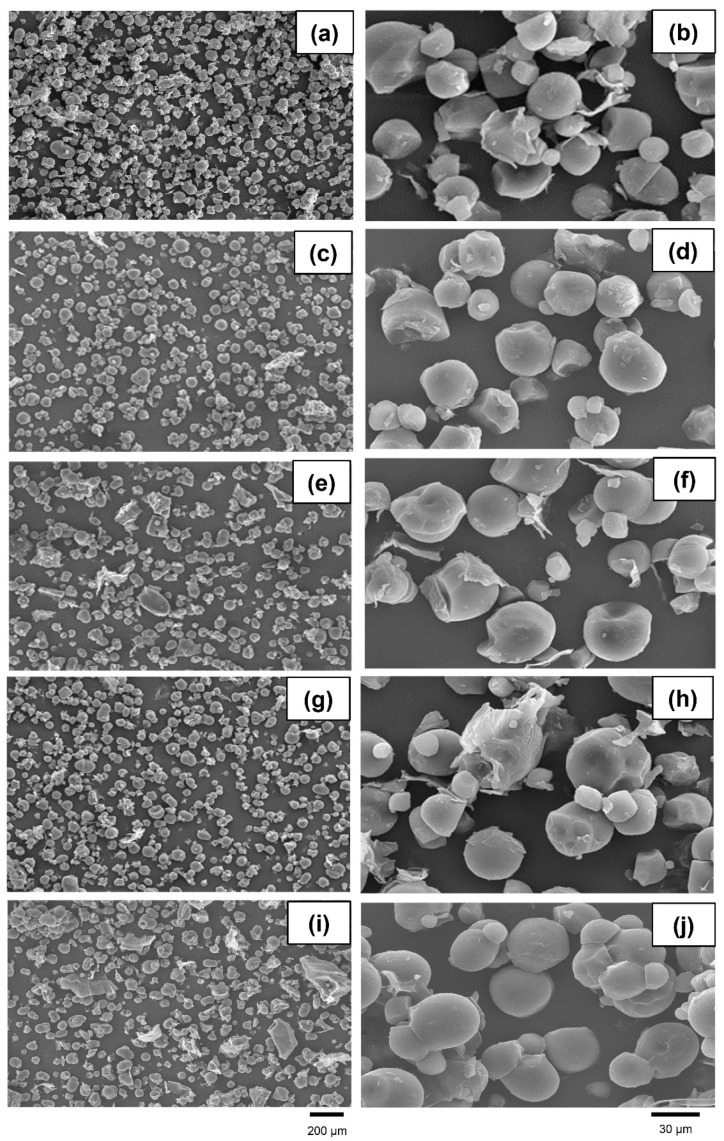
Microstructures of unprocessed (**a**,**b**), extruded (**c**,**d**), microwave-irradiated (**e**,**f**), gamma-irradiated (**g**,**h**), and NaOH-treated (**i**,**j**) cassava chips. Magnifications of photographs were recorded at 250× (**left**) and 1500× (**right**).

Minor changes in microstructures were observed across the five treatments (Figure 3). At a lower magnification, some irregular and unevenly distributed samples were found in the microwave-irradiated and NaOH-treated cassava chips, while a spherical shape with evenly distributed samples was prominently observed in the other remaining treatments. At a higher magnification, the unprocessed, extruded, and microwave-irradiated cassava chips had similar general features. Disruptions causing rough, laminated, and aggregated features were mainly observed in the gamma-irradiated and NaOH-treated groups.

### 3.4. In Vitro Nutrient Digestibility

#### 3.4.1. Nile Tilapia and Broiler Models

**Figure 4:**
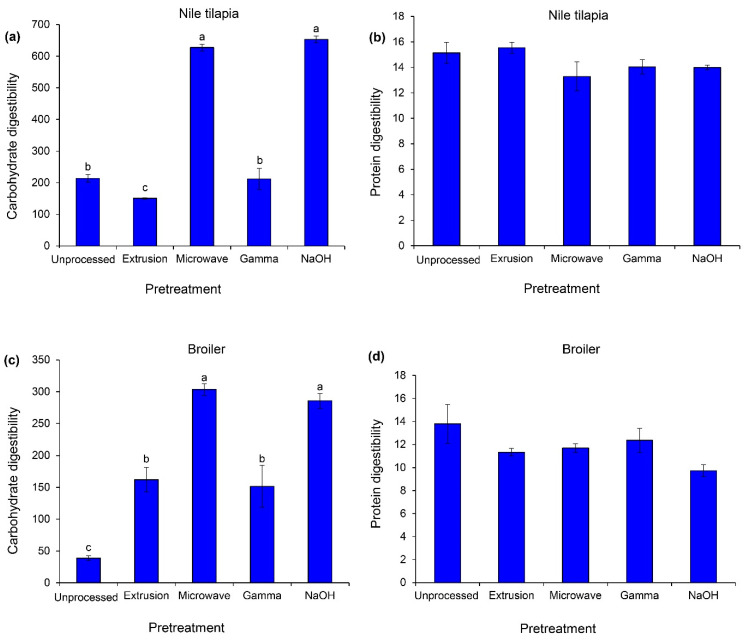
In vitro digestibility of carbohydrate (µmol maltose g sample^−1^, **left**) and protein (mmol *DL*-alanine equivalent g sample^−1^, **right**) of unprocessed and pretreated cassava chips. The sources of enzymes for in vitro testing were Nile tilapia (**a**,**b**) and broiler (**c**,**d**). Data are expressed as means ± SEM (*n* = 3). Significant differences between treatments are indicated by different superscripts (*p* < 0.05).

The pretreatment methods had significant effects on carbohydrate digestibility in Nile tilapia and broiler (*p* < 0.05, Figure 4), while unchanged protein digestibility was observed in both groups of animals (*p* > 0.05). In Nile tilapia, only microwave irradiation and NaOH treatments dramatically improved carbohydrate digestibility (*p* < 0.05). For broiler, both pretreatment methods increased approximately carbohydrate digestibility by five-fold, while an approximately two-fold increase was observed in the extruded and gamma-irradiated groups relative to the unprocessed cassava chips (*p* < 0.05).

#### 3.4.2. Ruminant Model

Varying pretreatment methods had no effects on the COMS, DOM, and ME (*p* > 0.05, Table 5). The observed values were within the ranges of 95.1–96.3%, 90.0–91.2%, and 13.4–13.5 MJ kg^−1^, respectively.

## 4. Discussion

### 4.1. Chemical Compositions of Pretreated Cassava Chips

Physical pretreatments significantly affected all proximate chemical compositions. These changes were also confirmed by the altered nineteen bands of the FTIR spectra within a range of 4000 to 400 cm^−1^. An improved CP content was observed in extruded cassava chips, while the pretreatments by microwave irradiation and NaOH hydrolysis provided the opposite results. It is possible that the covalent cross-linkages of protein and other N-containing compounds contribute to higher portions of molecular weight aggregates [42]. In addition, CP is the result of the multiplication of the N content in a sample by 6.25 or any other specific correction factor. Thus, the effect of extrusion can be derived from the reduction in other components in the evaluated samples, which increased the N content and, therefore, the CP content [17]. In contrast, the opposite direction of CP contents was observed in microwave-irradiated and NaOH-treated cassava chips. This is due to the high temperatures and alkaline treatments that break the raw material architecture, resulting in a significant loss of nutrients.

A significant reduction in EE content was affected by three alternative pretreatments, namely, extrusion, microwave irradiation, and NaOH hydrolysis. This loss may result from the formation of free radicals, enabling the molecules to react with conjugated systems and free radicals, which are often considered initiators of lipid oxidation. Moreover, irradiation can cause significant changes in lipid content and fatty acid profiles [43]. The varying exposure conditions in the current study may reduce undesirable effects.

Fluctuations in the amounts of NDF (which includes cellulose, hemicellulose, and lignin) and ADF (which includes cellulose and lignin) were observed. As compared to unprocessed cassava chips, four alternative pretreatments decreased the amount of cellulose and lignin significantly, while only gamma irradiation and NaOH reduced hemicellulose, indicating a higher formation of hemicellulose while cellulose and lignin degraded. Since the extrusion expanded NDF dramatically, this disruption still decreased the amounts of NFC in this treatment.

Ash contents tended to increase in all physical pretreatments, although insignificant for gamma irradiation. A chelating reaction can cause an ash increase in soaked or irradiated samples [44]. For the NaOH group, inorganic components from NaOH directly provide minerals, so the ash content can increase [45]. The changes in these nutritional contents significantly altered the DM, OM, and GE. In addition, NaOH drastically changed the nutritional constituents, such as proteins, lipids, and the principal component of starch [46], leading to a significantly reduced GE.

### 4.2. Physicochemical Properties of Pretreated Cassava Chips

Directly adding NaOH caused a significant increase in overall alkalinity. The release of hydroxyl groups from lignocellulosic degradation can also increase the pH value in microwave-irradiated samples [47]. On the other hand, the breakdown of macromolecules into smaller molecules provides acidic carboxyl groups [17,18]. An improved water-favoring capacity was found in extruded and NaOH-treated cassava chips, while a decreased WAC was found in the extruded samples. This characteristic may be associated with a hydrolytic capacity of digestive enzymes in utilizing this feed ingredient. The differences in nutritional constituents, especially hydrophilic residue containing several hydroxyl groups, polarizes them, which again is liable for water absorption [48].

Since cassava chips contain a large amount of NFC, the thermal response from the current study indicates the gelatinization characteristics of starch. At lower temperatures (peak I), similar thermal characteristics were observed, indicating no significant effects from pretreatments. However, some improvements, as indicated by a lowered ΔH, were observed. This indicates a molecular transformation that reflects the energy required for gelatinization and the destruction of the starch double-helix during gelatinization. At higher temperatures (peak II), significance in all thermal parameters (T_o_, T_p_, T_c_, and T_c_ − T_o_) was observed, indicating a change in complexation between amylose and other substances [49]. Therefore, the absence of peak II in the NaOH-treated sample might be associated with the destruction of the complex. Followed by a microwave irradiation, this method reduced ΔH significantly, indicating the partial destruction of the complex. For other pretreatment methods, some decreases in thermal parameters were observed. It is possible from the enfeebled granule of starch that affects gelatinization temperature [50]. The shift in each thermal parameter caused a significant change in T_c_ − T_o_, indicating heterogeneity in the form of cleaved polymers after pretreatments [51].

An X-ray diffraction pattern analysis was performed to investigate the crystal structure of the unprocessed and pretreated cassava chips. The characteristic diffraction peaks of cassava appeared at the angles of 15.1, 17.1, 18.0, and 26.6° (2θ), corresponding to the C-type mixed crystal structure between the B-type (granule core) and A-type (peripheral regions of the granule) [52]. In general, tapioca starch granules are predominantly spherical and have a smooth surface. However, the pretreatment of starch samples led to defect accumulation in the crystal structure, thus disrupting the double helices of amylopectin and single helices of amylase due to the rupture of hydrogen or glycosidic bonds [53]. Although the pretreatment of the cassava pieces resulted in slight changes in the structure of the starch granules, however, the pretreatment protocols from the current study did not affect the crystal structure of the starch that was observed via XRD. These results match with the microstructure observed via SEM. Some changes in the granule morphology induced by gelatinization were observed for the pretreated samples. A strong disruption was found in the gamma-irradiated and NaOH-treated groups, whereas the unprocessed, extruded, and microwave-irradiated cassava chips had similar general features.

### 4.3. In Vitro Digestibility Screening in Three Animal Models

Among four alternative methods, NaOH hydrolysis and microwave irradiation provided the highest in vitro carbohydrate digestibility, using digestive enzymes from Nile tilapia and broiler. However, a relatively high proportion and concentration of NaOH in the present study, based on the application in ruminant, may cause adverse effects in monogastric animals. The optimization of NaOH for feedstuff pretreatment should be investigated in these animal groups. For microwave irradiation, this pretreatment method improved the bioavailability of nutrients in several studies [17,18,54]. This pretreatment may improve the overall nutritive profile and physicochemical changes of cassava chips in the aspect of enhanced enzymatic hydrolysis. For broiler, both pretreatments followed by extrusion and gamma irradiation are also suitable. Practically, the extrusion of feed ingredients during pellet or crumble feed production could improve feed efficiency in broiler relative to mash feed.

For the ruminant model, the pepsin-cellulase technique has been used to estimate the digestibility of forages. This method provides a good correlation with the rumen fluid method, making it a good alternative method [55]. Based on our investigations in the current study, there were no differences in the digestibility of the COMS, DOM, and ME between unprocessed and pretreated cassava chips. However, carbohydrate digestibility was not assessed in the current study, therefore different nutritive profiles and physicochemical changes may directly affect this nutrient and should be further investigated.

## 5. Conclusions

The proximate chemical composition, nutritive values, and physicochemical properties of cassava chips fluctuated due to physical pretreatments. Among the four alternative pretreatments, based on in vitro carbohydrate digestibility, microwave irradiation was found to be suitable for preparing cassava chips for Nile tilapia and broiler, while extrusion or gamma irradiation was considered a second suitable option for the latter group. However, these four methods did not alter the pepsin-cellulase digestibility values testing for the ruminant group. Our investigations from the current study support the use of pretreated cassava chips for feeding fish and broiler. Further research is needed to determine the impact of these treatments and their applicability in different animal production contexts.

## Figures and Tables

**Table 1:** The chemical compositions (of dry matter) of unprocessed (control) and pretreated cassava chips used in the experiment. The values were calculated from triplicate samples.

Chemical Composition	Unprocessed	Extrusion	Microwave	Gamma	NaOH	*p*-Value
Dry matter (%)	95.3 ± 0.1 ^a^	85.9 ± 0.1 ^e^	94.1 ± 0.1 ^c^	94.9 ± 0.1 ^b^	92.3 ± 0.1 ^d^	<0.001
Organic matter (%)	97.9 ± 0.1 ^a^	97.3 ± 0.1 ^b^	97.5 ± 0.1 ^b^	97.7 ± 0.1 ^ab^	95.3± 0.1 ^c^	<0.001
Crude protein (%)	3.57 ± 0.10 ^b^	5.86 ± 0.06 ^a^	3.27 ± 0.01 ^c^	3.54 ± 0.01 ^b^	3.33 ± 0.01 ^c^	<0.001
Ether extract (%)	0.51 ± 0.01 ^a^	0.43 ± 0.01 ^b^	0.27 ± 0.01 ^c^	0.48 ± 0.02 ^a^	0.25 ± 0.01 ^c^	<0.001
Neutral detergent fiber (%)	14.7 ± 0.2 ^c^	28.7 ± 0.5 ^a^	16.6 ± 0.1 ^b^	7.6 ± 0.1 ^e^	11.1 ± 0.1 ^d^	<0.001
Acid detergent fiber (%)	6.82 ± 0.11 ^a^	6.65 ± 0.09 ^b^	5.89 ± 0.11 ^cd^	5.71 ± 0.07 ^d^	6.53 ± 0.04 ^c^	<0.001
Ash (%)	2.27 ± 0.01 ^d^	2.69 ± 0.03 ^b^	2.53 ± 0.02 ^c^	2.40 ± 0.04 ^cd^	5.08 ± 0.05 ^a^	<0.001
Non-fiber carbohydrate (%)	79.6 ± 0.2 ^c^	66.4 ± 0.5 ^e^	78.3 ± 0.1 ^d^	86.4 ± 0.01 ^a^	81.1 ± 0.1 ^b^	<0.001
Gross energy (kcal kg^−1^)	3972 ± 33 ^b^	4139 ± 7 ^a^	4030 ± 37 ^b^	3993 ± 8 ^b^	3840 ± 8 ^c^	0.002

^a–e^ Means in the same row with different superscripts indicate significant differences (*p* < 0.05).

**Table 2:** Tentative assignment of FTIR spectral peaks found in unprocessed (control) and pretreated cassava chips.

Wavenumber (cm^−1^)	Tentative Band Assignment	Macromolecule	References
2924	*v*_as_ (CH_2_) stretching of methylene	Lipid	[30,31]
1637	*v*_s_ (C=O) stretching of amide I	Protein	[32]
1439	δ_as_ (CH_2_) bending of methyl	Lipid	[30,31]
	δ_as_ (CH_3_) bending of methyl	Protein	[33]
1412	δ_as_ (CH_2_) bending of methyl	Lipid	[30,31]
	δ_as_ (CH_3_) bending of methyl	Protein	[33]
	*v*_s_ (C=O) stretching vibrations of carboxylate	Carbohydrate	[34]
1373	*v*_s_ (COO-) stretching of amino acid salt	Protein	[35]
	*v*_s_ (C=O) stretching vibrations of carboxylate	Carbohydrate	[34]
1336	*v*_s_ (C=O) stretching vibrations of carboxylate	Carbohydrate	[34]
1242	N(C=O) stretching and (C-OH) bending of deprotonated amino acid	Protein	[36]
1151	*v* (C-O-C) stretching of polysaccharide	Carbohydrate	[37]
1053	*v* (C-O-C) stretching of polysaccharide	Carbohydrate	[37]
997	*v* (C-O-C) stretching of polysaccharide	Carbohydrate	[37]
929	C=C bending of alkene	Alkene	[35]
860	*v*_as_ (PO_4_^3−^) P-O asymmetric stretching of lipids	Lipid	[38]
765	δ (CO_3_^2−^) Out of O-C=O bending of oxalate	Inorganic	[33]
709	(CH^2−^), C-H rocking of lipids	Lipid	[31]
603	C-Br stretching of halo compound	Inorganic	[39]
578	C-Br stretching of halo compound	Inorganic	[39]
522	N-C=O of amides	Amides	[40]
484	*v*_4_ (PO_4_^3−^) P-O stretching of tetrahedral inorganic molecules	Inorganic	[41]
434	*v*_4_ (PO_4_^3−^) P-O stretching of tetrahedral inorganic molecules	Inorganic	[41]

**Table 3:** pH, water solubility, and water absorption capacity (WAC) of unprocessed (control) and pretreated cassava chips used in the experiment. The values were calculated from triplicate samples.

Item	Unprocessed	Extrusion	Microwave	Gamma	NaOH	*p*-Value
pH	6.1 ± 0.1 ^c^	6.1 ± 0.1 ^c^	6.2 ± 0.1 ^b^	6.0 ± 0.1 ^d^	10.4 ± 0.1 ^a^	<0.001
Water solubility (%)	9.0 ± 0.2 ^c^	19.9 ± 0.4 ^a^	10.1 ± 2.6 ^c^	9.7 ± 0.4 ^c^	14.4 ± 0.1 ^b^	<0.001
WAC (g water g feed^−1^)	0.30 ± 0.01 ^a^	0.26 ± 0.01 ^b^	0.32 ± 0.01 ^a^	0.28 ± 0.02 ^ab^	0.32 ± 0.01 ^a^	0.020

^a–d^ Means in the same row with different superscripts indicate significant differences (*p* < 0.05).

**Table 4:** Thermal transition properties of unprocessed (control) and pretreated cassava chips used in the experiment. The values were calculated from triplicate samples.

Thermal Parameter	Unprocessed	Extrusion	Microwave	Gamma	NaOH	*p*-Value
Peak I						
T_o_ (°C)	46.0 ± 0.6	46.1 ± 0.8	45.3 ± 2.8	46.9 ± 0.9	47.0 ± 0.6	0.901
T_p_ (°C)	91.2 ± 1.4	90.1 ± 0.8	91.6 ± 1.6	91.2 ± 1.0	89.7 ± 1.5	0.798
T_c_ (°C)	147 ± 2	150 ± 1	151 ± 3	153 ± 2	157 ± 3	0.100
T_c_ − T_o_ (°C)	101 ± 1	104 ± 2	106 ± 2	106 ± 3	110 ± 3	0.169
∆H (J g^−1^)	225 ± 10 ^a^	181 ± 4 ^c^	211 ± 6 ^ab^	193 ± 0.3 ^bc^	206 ± 4 ^b^	0.003
Peak II						
T_o_ (°C)	266 ± 1 ^a^	265 ± 1 ^a^	265 ± 1 ^a^	259 ± 1 ^b^	–	<0.001
T_p_ (°C)	274 ± 1 ^a^	271 ± 1 ^b^	273 ± 1 ^a^	270 ± 1 ^c^	–	<0.001
T_c_ (°C)	280 ± 1 ^a^	277 ± 1 ^c^	281 ± 1 ^a^	278 ± 1 ^b^	–	<0.001
T_c_ − T_o_ (°C)	14.9 ± 0.2 ^b^	12.1 ± 0.1 ^c^	15.2 ± 0.8 ^b^	18.2 ± 0.3 ^a^	–	<0.001
∆H (J g^−1^)	4.55 ± 0.07 ^a^	4.93 ± 0.14 ^a^	3.86 ± 0.20 ^b^	4.99 ± 0.24 ^a^	–	<0.001

T_o_, onset temperature; T_p_, peak temperature; T_c_, conclusion temperature; T_c_ − T_o_, melting temperature range, ∆H, enthalpy. ^a–c^ Means in the same row with different superscripts indicate significant differences (*p* < 0.05).

**Table 5:** The pepsin-cellulase digestibility (of dry matter) of unprocessed (control) and pretreated cassava chips for the ruminant model.

Item	Unprocessed	Extrusion	Microwave	Gamma	NaOH	*p*-Value
COMS (%)	95.1 ± 1.0	95.2 ± 0.7	96.3 ± 0.8	95.1 ± 0.1	95.8 ± 0.6	0.722
DOM (%)	90.1 ± 1.0	90.2 ± 0.7	91.2 ± 0.8	90.0 ± 0.1	90.8 ± 0.6	0.720
ME (MJ kg^−1^)	13.4 ± 0.2	13.4 ± 0.1	13.5 ± 0.1	13.4 ± 0.02	13.4 ± 0.1	0.890

COMS, cellulase organic matter solubility; DOM, digestible organic matter; ME, metabolizable energy.

## Data Availability

The data presented in this study are available upon request from the corresponding author.

## References

[1] Morgan N.K., Choct M. (2016). Cassava: Nutrient composition and nutritive value in poultry diets. Anim. Nutr..

[2] International Institute of Tropical Agriculture (IITA) (1990). Cassava in Tropical Agriculture: A Practical Manual.

[3] Bizzuti B.E., de Abreu Faria L., da Costa W.S., Lima P.M.T., Ovani V.S., Krüger A.M., Louvandini H., Abdalla A.L. (2021). Potential use of cassava by-product as ruminant feed. Trop. Anim. Health Prod..

[4] Mahanama D., Radampola K., Heenkenda E. (2021). Effect of cassava starch sources on growth and feed utilization of Nile tilapia fingerlings (*Oreochromis niloticus*) reared under two dietary protein levels. Aquac. Stud..

[5] Yadav S., Mishra B., Jha R. (2019). Cassava (*Manihot esculenta*) root chips inclusion in the diets of broiler chickens: Effects on growth performance, ileal histomorphology, and cecal volatile fatty acid production. Poult. Sci..

[6] Conde L.A., Kebede B., Leong S.Y., Oey I. (2022). Changes in starch in vitro digestibility and properties of cassava flour due to pulsed electric field processing. Foods.

[7] Kantho U., Juttupornpong S., Howeler R.H. (2002). Clean cassava chips for animal feeding in Thailand. Cassava Research and Development in Asia: Exploring New Opportunities for an Ancient Crop.

[8] Truelock C.N., Tokach M.D., Stark C.R., Paulk C.B. (2020). Pelleting and starch characteristics of diets containing different corn varieties. Transl. Anim. Sci..

[9] Hassan A.B., Pawelzik E., von Hoersten D. (2021). Effect of microwave heating on the physiochemical characteristics, colour and pasting properties of corn (*Zea mays* L.) grain. LWT—Food Sci. Technol..

[10] Manupriya B.R., Lathika, Somashekarappa H.M., Patil S.L., Shenoy K.B. (2020). Study of gamma irradiation effects on the physico-chemical properties of wheat flour (*Triticum aestivum* L.). Radiat. Phys. Chem..

[11] Sansuwan K., Kovitvadhi S., Thongprajukaew K., Ozorio R.O.A., Somsueb P., Kovitvadhi U. (2017). Microwave irradiation and pelleting method affected feed chemical composition and growth performance and feed utilization of sex-reversed Nile tilapia, *Oreochromis niloticus* (L.). Aquac. Res..

[12] Zhu L., Yu B., Chen H., Yu J., Yan H., Luo Y., He J., Huang Z., Zheng P., Mao X. (2022). Comparisons of the micronization, steam explosion, and gamma irradiation treatment on chemical composition, structure, physicochemical properties, and in vitro digestibility of dietary fiber from soybean hulls. Food Chem..

[13] Gonzalez-Rivas P.A., DiGiacomo K., Giraldo P.A., Leury B.J., Cottrell J.J., Dunshea F.R. (2017). Reducing rumen starch fermentation of wheat with three percent sodium hydroxide has the potential to ameliorate the effect of heat stress in grain-fed wethers. J. Anim. Sci..

[14] Abraham A., Mathew A., Park H., Choi O., Sindhu R., Parameswaran B., Pandey A., Park J.H., Sang B.I. (2020). Pretreatment strategies for enhanced biogas production from lignocellulosic biomass. Bioresour. Technol..

[15] Li Y., Jin Y., Li J., Li H., Yu Z., Nie Y. (2017). Effects of thermal pretreatment on degradation kinetics of organics during kitchen waste anaerobic digestion. Energy.

[16] Zhu F. (2018). Relationships between amylopectin internal molecular structure and physicochemical properties of starch. Trends Food Sci. Technol..

[17] Hahor W., Thongprajukaew K., Nuntapong N., Saekhow S., Rungruangsak-Torrissen K., Dumrongrittamatt T., Phonchai A. (2022). Partial pretreatment of ingredient mixture effectively improved feed chemical composition, physicochemical properties and in vitro digestibility. Anim. Feed Sci. Technol..

[18] Thongprajukaew K., Rodjaroen K., Tantikitti C., Kovitvadhi U. (2015). Physicochemical modifications of dietary palm kernel meal affect growth and feed utilization of Nile tilapia (*Oreochromis niloticus*). Anim. Feed Sci. Technol..

[19] Zhou P., Theil P.K., Wu D., Knudsen K.E.B. (2018). In vitro digestion methods to characterize the physicochemical properties of diets varying in dietary fibre source and content. Anim. Feed Sci. Technol..

[20] Sutthinon P., Hahor W., Chumchuen K., Thongprajukaew K. (2023). Ontogenetic development of digestive enzymes and in vitro digestibility of spotted Babylon (*Babylonia areolata*) veligers. Aquac. Rep..

[21] Shawrang P., Nikkhah A., Zare-Shahneh A., Sadeghi A.A., Raisali G., Moradi-Shahrebabak M. (2008). Effects of gamma irradiation on chemical composition and ruminal protein degradation of canola meal. Radiat. Phys. Chem..

[22] De Campeneere S., De Brabander D.L., Vanacker J.M. (2006). Milk urea concentration as affected by the roughage type offered to dairy cattle. Livest. Sci..

[23] AOAC (1990). Official Methods of Analysis.

[24] Van Soest P.J., Robertson J.B., Lewis B.A. (1991). Methods for dietary fibre, neutral detergent fibre and non-starch polysaccharides in relation to animal nutrition. J. Dairy Sci..

[25] Sokhey A.S., Chinnaswamy R. (1993). Chemical and molecular properties of irradiated starch extrudates. Cereal Chem..

[26] Chung B.Y., Firth A.E., Atkins J.F. (2010). Frameshifting in alphaviruses: A diversity of 3′ stimulatory structures. J. Mol. Biol..

[27] Jitngarmkusol S., Hongsuwankul J., Tananuwong K. (2008). Chemical compositions, functional properties, and microstructure of defatted macadamia flours. Food Chem..

[28] Thongprajukaew K., Kovitvadhi U., Kovitvadhi S., Somsueb P., Rungruangsak-Torrissen K. (2011). Effects of different modified diets on growth, digestive enzyme activities and muscle compositions in juvenile Siamese fighting fish (*Betta splendens* Regan, 1910). Aquaculture.

[29] De Boever J.L., Cottyn B.G., Buysse F.X., Wainman F.W., Vanacker J.M. (1986). The use of an enzymatic technique to predict digestibility, metabolizable and net energy of compound feedstuff: For ruminant. Anim. Feed Sci. Technol..

[30] Lewis R.N., McElhaney R.N., Mantsch H.H., Chapman D. (1996). Fourier transform infrared spectroscopy in the study of hydrated lipids and lipid bilayer membranes. Infrared Spectroscopy of Biomolecules.

[31] Stuart F.I. (1997). Supply-chain strategy: Organizational influence through supplier alliances. Br. J. Manag..

[32] Dean M., Raats M.M., Shepherd R. (2008). Moral concerns and consumer choice of fresh and processed organic foods. J. Appl. Soc. Psych..

[33] Giordano M., Kansiz M., Heraud P., Beardall J., Wood B., McNaughton D. (2001). Fourier transform infrared spectroscopy as a novel tool to investigate changes in intracellular macromolecular pools in the marine microalga *Chaetoceros muellerii* (Bacillariophyceae). J. Phycol..

[34] Falkeborg M., Cheong L.Z., Gianfico C., Sztukiel K.M., Kristensen K., Glasius M., Xu X., Guo Z. (2014). Alginate oligosaccharides: Enzymatic preparation and antioxidant property evaluation. Food Chem..

[35] Guzman J., Esmail R., Karjalainen K., Malmivaara A., Irvin E., Bombardier C. (2001). Multidisciplinary rehabilitation for chronic low back pain: Systematic review. BMJ.

[36] Li W., Raoult D., Fournier P.E. (2009). Bacterial strain typing in the genomic era. FEMS Microbiol. Rev..

[37] Brandenburg K., Seydel U., Mantsch H.H., Chapman D. (1996). Fourier transform infrared spectroscopy of cell surface polysaccharides. Infrared Spectroscopy of Biomolecules.

[38] Dean A.P., Martin M.C., Sigee D.C. (2007). Resolution of codominant phytoplankton species in a eutrophic lake using synchrotron-based Fourier transform infrared spectroscopy. Phycologia.

[39] Wong P., Goldstein S., Grekin R., Godwin A., Pivik C., Riga B. (1993). Distinct infrared spectroscopic pattern of human basal cell carcinoma of the skin. Cancer Res..

[40] Maquelin K., Kirschner C., Choo-Smith L.P., Braak N.V.D., Ph Endtz H., Naumann D., Puppels G.J. (2002). Identification of medically relevant microorganisms by vibrational spectroscopy. J. Microbiol. Methods.

[41] Benning L.G., Phoenix V.R., Yee N., Tobin M.J. (2004). Molecular characterization of *Cyanobacterial silification* using synchrotron infrared micro-spectroscopy. Geochim. Cosmochim. Acta.

[42] Sadeghi A.A., Shawrang P. (2006). Effects of microwave irradiation on ruminal degradability and in vitro digestibility of canola meal. Anim. Feed Sci. Technol..

[43] Rodjaroen S., Thongprajukaew K., Saekhow S. (2018). Physical pretreatments for improving nutritive value of cyanobacterial cells. Chiang Mai J. Sci..

[44] Chumwaengwapee S., Soontornchai S., Thongprajukeaw K. (2013). Improving chemical composition, physicochemical properties, and in vitro carbohydrate digestibility of fish coconut meal. ScienceAsia.

[45] Offner A., Bach A., Sauvant D. (2003). Quantitative review of in situ starch degradation in the rumen. Anim. Feed Sci. Technol..

[46] Palacios-Fonseca A.J., Castro-Rosas J., Gomez-Aldapa C.A., Tovar-Benitez T., Millan-Malo B.M., Real del A., Rodriguez-Garcia M.E. (2013). Effect of the alkaline and acid treatments on the physicochemical properties of corn starch. J. Food Sci..

[47] Thongprajukaew K., Yawang P., Dudae L., Bilanglod H., Dumrongrittamatt T., Tantikitti C., Kovitvadhi U. (2013). Physical modification of palm kernel meal improved available carbohydrate, physicochemical properties and in vitro digestibility in economic freshwater fish. J. Sci. Food Agric..

[48] Rashid M.M., Mohammed K., Mesfer A., Naseem H., Danish M. (2015). Hydrogen production by water electrolysis: A review of alkaline water electrolysis, PEM water electrolysis and high temperature water electrolysis. Int. J. Eng. Adv. Technol..

[49] Caetano M., Goulart R.S., Rizzo P.M., Silva S.L., Drouillard J.S., Leme P.R., Lanna D.P.D. (2019). Impact of flint corn processing method and dietary starch concentration on finishing performance of Nellore bulls. Anim. Feed Sci. Technol..

[50] MacArthur L.A., Appolonia B.L. (1984). Gamma radiation of wheat. II. Effects of low-dosage radiations on starch properties. Cereal Chem..

[51] Jualaong S., Songnui A., Thongprajukaew K., Ninwat S., Khwanmaung S., Hahor W., Khunsaeng P., Kanghae H. (2019). Optimal salinity for head-starting northern river terrapins (*Batagur baska* Gray, 1831). Animals.

[52] Zhao A.-Q., Yu L., Yang M., Wang C.-J., Wang M.-M., Bai X. (2018). Effects of the combination of freeze-thawing and enzymatic hydrolysis on the microstructure and physicochemical properties of porous corn starch. Food Hydrocoll..

[53] Dome K., Podgorbunskikh E., Bychkov A., Lomovsky O. (2020). Changes in the crystallinity degree of starch having different types of crystal structure after mechanical pretreatment. Polymers.

[54] Zeller E., Schollenberger M., Kühn I., Rodehutscord M. (2015). Effect of diets containing enzyme supplements and microwave-treated or untreated wheat on inositol phosphates in the small intestine of broilers. Anim. Feed Sci. Technol..

[55] Nousiainen J., Rinne M., Hellämäki M., Huhtanen P. (2003). Prediction of the digestibility of the primary growth of grass silages harvested at different stages of maturity from chemical composition and pepsin-cellulase solubility. Anim. Feed Sci. Technol..

